# Risk and Risk Factors Associated With Recurrent Venous Thromboembolism Following Surgery in Patients With History of Venous Thromboembolism

**DOI:** 10.1001/jamanetworkopen.2019.3690

**Published:** 2019-05-10

**Authors:** Banne Nemeth, Willem M. Lijfering, Rob G. H. H. Nelissen, Inger B. Schipper, Frits R. Rosendaal, Saskia le Cessie, Suzanne C. Cannegieter

**Affiliations:** 1 Department of Clinical Epidemiology, Leiden University Medical Center, Leiden, the Netherlands; 2 Department of Orthopedics, Leiden University Medical Center, Leiden, the Netherlands; 3 Department of Trauma Surgery, Leiden University Medical Center, Leiden, the Netherlands; 4 Medical Statistics, Department of Biomedical Datascience, Leiden University Medical Center, Leiden, the Netherlands; 5 Department of Thrombosis and Haemostasis, Leiden University Medical Center, Leiden, the Netherlands

## Abstract

**Importance:**

The size of the risk of recurrent venous thromboembolism (VTE) after surgery in patients with a history of VTE is not well known.

**Objectives:**

To estimate the risk of and to identify the factors associated with recurrent VTE in patients undergoing surgery who have a history of VTE.

**Design, Setting, and Participants:**

This population-based, follow-up cohort study includes patients with VTE who participated in the Multiple Environment and Genetic Assessment (MEGA) study. Original data were collected from March 1999 to April 2010. Data analysis began in June 1999 and ended in April 2010.

**Exposures:**

Surgery following a first VTE.

**Main Outcomes and Measurements:**

Kaplan-Meier analyses were used to estimate cumulative incidences of recurrent VTE. Cox regression with a time-dependent covariate (surgery) was used to calculate the hazard ratio (HR) for developing recurrent VTE after surgery compared with no surgery.

**Results:**

Overall, 3741 patients (mean [SD] age, 48.4 [12.8] years; 2020 [54.0%] women) with a history of VTE were included in the analysis, amounting to 18 899 person-years, with a median (interquartile range) follow-up of 5.7 (3.0-7.2) years. Of the 3741 patients, 580 (15.5%) underwent surgery and 601 (16.1%) developed a recurrent thrombotic event. The 1-month cumulative incidence of recurrent VTE for all surgery types was 2.1% (95% CI, 1.2%-3.6%), which increased to 3.3% (95% CI, 2.1%-5.1%) at 3 months and 4.6% (95% CI, 3.1%-6.6%) at 6 months. At 6 months, risk of recurrent VTE ranged from 2.3% to 9.3%, depending on surgery type. In addition to surgery type, factor V Leiden mutation (HR, 3.4; 95% CI, 1.6-7.4) and male sex (HR, 2.7; 95% CI, 1.3-5.8) were associated with increased risk of recurrent VTE.

**Conclusions and Relevance:**

Surgery was associated with an increased risk of recurrent VTE in patients with a history of VTE; risk remained high for up to 6 months after the procedure. This study suggests that high-risk individuals may be identified based on surgery type, sex, and the presence of factor V Leiden mutation. These findings stress the need for revision of the current thromboprophylactic approach to prevent recurrence in these patients.

## Key Points

**Question:**

What is the risk of recurrent venous thromboembolism (VTE) following surgery in patients with a history of VTE?

**Findings:**

In this longitudinal follow-up cohort study, 3741 patients with a history of VTE were evaluated for the development of recurrence and 15.5% underwent surgery. Cumulative incidences of VTE recurrence were 2.1% at 1 month, 3.3% at 3 months, and 4.6% at 6 months; in addition to surgery type (eg, cancer-related surgery), male sex and factor V Leiden mutation were associated with a higher risk of recurrence.

**Meaning:**

Patients with a history of VTE who undergo surgery may have a high recurrence risk, a finding that challenges the current thromboprophylactic strategy for these patients.

## Teaser

This cohort study estimates the risk of and factors associated with recurrent venous thromboembolism in patients undergoing surgery who have a history of venous thromboembolism.

## Introduction

Surgery is a major risk factor for the development of venous thromboembolism (VTE), encompassing both deep vein thrombosis and pulmonary embolism.^1^ For this reason, routine thromboprophylaxis therapy is strongly recommended for high-risk individuals undergoing general surgery and for all patients who undergo major orthopedic surgery, unless contraindicated.^1,2^ Although the risk of developing a first VTE after surgery has been studied extensively, there are few studies that evaluate the size of the recurrence risk in patients with a history of VTE who undergo surgery. Several studies^3,4,5^ showed an increased risk in patients with a history of VTE who underwent surgery compared with individuals without a history of VTE. Yet, to our knowledge, only a single study^6^ addressed whether patients with a previous VTE are at increased risk after surgery compared with patients with VTE who did not undergo surgery. This is a more clinically relevant comparison because, if this is the case, additional thromboprophylactic measures are asked for. This study found a 3-fold increased risk of developing recurrence up to 92 days postdismissal.^6^ However, the authors were not able to distinguish between various types of surgery, and more importantly, absolute risks could not be determined.

It is advised that clinicians assess an individual’s thrombosis risk by using risk scores, such as the Caprini score,^4,7^ to evaluate risk factors of VTE in all patients undergoing surgery.^1,8,9^ Individuals with a history of VTE are almost always classified as being at moderate to high risk. Consequently, thromboprophylactic therapy is indicated for most of these patients (unless there is also a high risk of major bleeding) during hospitalization following surgery.^1,9^ However, it is not clear if this treatment sufficiently lowers the risk among this high-risk group. Furthermore, risks may differ between individuals, depending on surgery type and other clinical or laboratory risk factors. For example, no differentiation is currently made with respect to the dosage or duration of thromboprophylaxis in patients at high risk.

Because these data are essential to guide physicians in thromboprophylaxis management following surgery, we set out to determine the size of the risk of recurrent VTE in patients with a history of VTE who undergo surgery. In addition, we identified factors associated with recurrence in these patients.

## Methods

### Study Design

For this study, data from the Multiple Environment and Genetic Assessment (MEGA) follow-up study were used, details of which have been published previously.^10,11,12^ Briefly, the MEGA study is a large population-based case-control study of the etiology of VTE and includes 4956 individuals with VTE and 6297 control participants.^13^ Unselected patients aged 18 to 70 years with a confirmed pulmonary embolism or deep vein thrombosis were recruited from 6 anticoagulation clinics in the Netherlands between March 1999 and August 2004. The trial protocol is available in Supplement 1.Subsequently, all patients with a first VTE who provided written informed consent to participate in the MEGA follow-up study were evaluated for recurrent VTE until April 2010. Initial information on the recurrent event was collected by means of a short questionnaire or telephone interview. Further detailed information about the recurrent event was retrieved from questionnaires, anticoagulation clinics, treating physicians, or cause of death statistics (vital status from the central Dutch Population Register).^12^ Recurrent events were adjudicated as certain or uncertain recurrent events^10^ to distinguish between genuinely new events and extensions of the first event. The decision rule for event classification is available in eMethods in Supplement 2. For the current analysis, only certain recurrent events were used to minimize misclassification. Patients with an uncertain event had a similar age and sex distribution (mean [SD] age, 50.1 [13.6] years; 107 (54.0%) women) compared with the study population.

All participants provided written informed consent. This study was approved by the Medical Ethical Committee of the Leiden University Medical Center in Leiden, the Netherlands. This study followed the Strengthening the Reporting of Observational Studies in Epidemiology (STROBE) reporting guideline for cohort studies.

### Data Collection and Surgery Exposure

After inclusion in the study, patients completed a questionnaire on putative risk factors of recurrent VTE, including age, sex, weight and height, and comorbidities. In 2011, participants of the MEGA study were linked to the Dutch Hospital Data registry.^14^ This registry provides nationwide electronic coverage of data on all hospital admissions since 1995. For each admission, information on dates of admission and discharge, diagnoses, and surgical procedures is available (coded according to the *International Classification of Diseases, Ninth Revision, Clinical Modification*). A previous study comparing a random sample of hospital admissions in the Dutch Hospital Data registry with information from hospital records^15^ showed that 99% of the personal, admission, and discharge data and 84% of the principal diagnosis data were correctly encoded. Individuals with information leading to more than 1 person (eg, twins) or to no one at all (eg, immigrants or visitors) were excluded. Of the 4956 MEGA participants with VTE, 4721 patients (95.3%) could be uniquely linked to the registry.

We collected information on all surgical procedures and operations for which patients were hospitalized for 1 or more days. We defined major surgical procedures (in terms of VTE risk) as those with an estimated duration longer than 30 minutes and minor surgical procedures as those with an estimated duration shorter than 30 minutes. The association of cancer-related surgery with recurrence risk was also studied.

### Statistical Analysis

**Figure 1.**
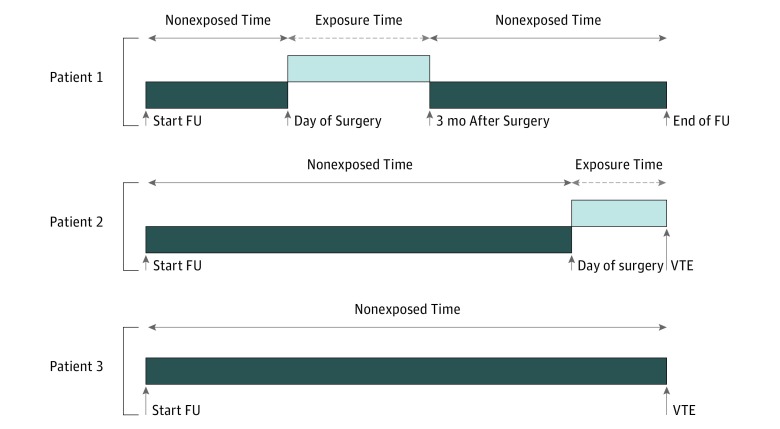
Visualization of Time-Dependent Analysis Exposure time denotes the time window of surgery exposure during which each individual was at risk of venous thromboembolism (VTE; 1 month, 3 months, 6 months, or 1 year). Three hypothetical patient pathways are presented. Patient 1 represents an individual who underwent surgery halfway through follow-up (FU) with no thrombotic event during exposure time. Patient 2 represents an individual who developed a thrombotic event within the surgery exposure time, and patient 3 represents an individual who had no surgery during FU but developed VTE.

Patient demographic characteristics were listed as means with standard deviations or numbers with percentages, depending on data type. Since we were interested in the risk of recurrent VTE after anticoagulation therapy for the first VTE had been stopped, follow-up time was calculated from the stop date of anticoagulation treatment after a patient’s first VTE until the end of study, death, recurrent event, or loss to follow-up, whichever occurred first. The window of exposure to surgical procedures during which an individual was at risk of VTE was defined as 3 months from the surgery date and later. The total follow-up time in which patients were not exposed to surgery was calculated as the total follow-up time minus surgery exposure time (Figure 1). Because it is unclear for how long the risk of recurrent VTE is increased after surgery, we varied the exposure time and considered 1-month, 3-month, 6-month, and 1-year windows as risk periods. For all analyses, we included only the first surgery exposure during follow-up, and patients were censored when they underwent a second surgery. As a sensitivity analysis, we did not censor these patients and also considered a second surgery as an exposure (eTable 1 in Supplement 2). Thus, patients could be exposed to multiple periods of increased risk (eg, first, second, and third surgery) during follow-up.

For the main outcome, we calculated the cumulative incidence of recurrent VTE over time for exposure to several types of surgery using life-table techniques (Kaplan-Meier). To compare with the cumulative incidence without surgery (ie, to show the excess recurrence risk after surgery), we estimated the expected cumulative recurrence risk for each patient who underwent surgery in the same period in the absence of surgery. These expected recurrence risks were obtained from a Kaplan-Meier curve of the total population, with follow-up time censored at time of surgery. A sensitivity analysis was performed with a landmark analysis for which we used the median time to surgery of 713 days. The risk of VTE in patients unexposed to surgery was calculated from this time point onwards.

Cox regression analysis with a time-dependent covariate (exposure time after surgery) was used to calculate hazard ratios (HRs) with 95% CIs for developing a recurrent VTE, adjusted for age and sex. In a restriction analysis, we excluded patients with a cancer diagnosis in the 5 years before their first VTE. Likewise, patients who developed cancer during follow-up were excluded in a second restriction analysis. An additional Cox regression analysis was performed to identify factors associated with recurrence, in which we adjusted for time between VTE and surgery (ie, follow-up began on day of surgery). The association with recurrent VTE was assessed for 10 potential or established prognostic determinants of recurrent VTE, including increasing age, male sex, non-O blood type, factor V Leiden mutation, prothrombin 20210A mutation, pulmonary embolism or deep vein thrombosis as a first event, obesity, self-reported comorbidity,^16,17^ provoked first venous thrombosis, and time elapsed since first VTE. We calculated HRs with 95% CIs for these factors and cumulative incidences for recurrence (Kaplan-Meier). There were no missing data for the main analysis; for the risk factor analysis, a complete case analysis was performed because some comorbidities were missing. All analyses were performed using SPSS software version 23.0 (IBM) and Stata Package SE version 14.0 (StataCorp).

## Results

### Study Population

Of the 4721 patients who could be linked to the Dutch Hospital Data registry, 371 did not consent to participate in the follow-up, resulting in 4350 total participants. In addition, 609 patients were excluded because they continued anticoagulation therapy after their first VTE throughout the follow-up period (eFigure in Supplement 2). Therefore, in total, 3741 patients were evaluated for a total of 18 899 person-years (median [IQR] follow-up, 5.7 [3.0-7.2] years). The mean (SD) age at start of follow-up (after first VTE) was 48.4 (12.8) years, and 2020 (54.0%) were women (Table 1). Overall, 601 patients (16.1%) developed a recurrent event. Most patients (2748 [82.9%]) had no major illnesses in their medical history at time of first VTE.

**Table 1.** Characteristics of Patients Included in the Multiple Environment and Genetic Assessment (MEGA) Follow-up Study

Characteristic	No. (%) (N = 3741)
Age, mean (SD), y	48.4 (12.8)
Women	2020 (54.0)
BMI, mean (SD)^a^	26.8 (14.0)
Comorbidity^b^	
No major illness	2748 (82.9)
Any major illness	569 (17.1)
COPD	204 (6.2)
Liver disease	18 (0.5)
Kidney disease	35 (1.1)
Rheumatoid arthritis	111 (3.4)
Multiple sclerosis	16 (0.5)
Heart failure	46 (1.4)
Hemorrhagic stroke	23 (0.7)
Arterial thrombosis	197 (5.3)
Myocardial infarction	94 (2.9)
Angina	47 (1.4)
Ischemic stroke	9 (0.3)
Transient ischemic attack	38 (1.2)
Peripheral vascular disease	41 (1.2)

Abbreviations: BMI, body mass index; COPD, chronic obstructive pulmonary disease.

^a^ Calculated as weight in kilograms divided by height in meters squared. Data missing for 326 patients.

^b^ Number total and any major illnesses missing for 424 patients.

### Surgical Procedures

In total, 580 patients (15.5%) had undergone 1 or more operations (808 total operations during the complete follow-up period) (eTable 2 in Supplement 2). Overall, 578 major operations and 230 minor operations were performed, of which 275 were orthopedic and 533 nonorthopedic. A detailed overview of all surgical procedures (including type of surgery) that were included in the analysis is given in eTable 3 in Supplement 2. Median (IQR) time to first surgery was 713 (252-1334) days.

### VTE Recurrence Risk

**Figure 2.**
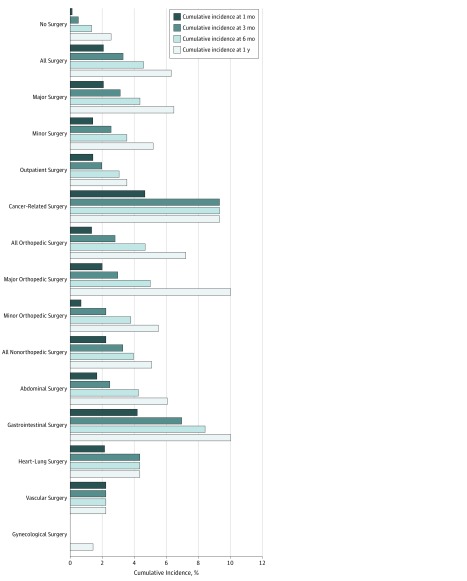
Absolute Risk of Recurrent Venous Thromboembolism After Surgery in Patients With a History of Venous Thromboembolism

Of all 580 patients who underwent a surgical procedure during follow-up, 13 patients developed a recurrent event within 1 month, 21 patients within 3 months, 30 patients within 6 months, and 38 patients within 12 months after surgery (ie, 38 events total). The cumulative incidence of recurrent VTE at 1 month was 2.1% (95% CI, 1.2%-3.6%), which increased to 3.3% (95% CI, 2.1%-5.1%) at 3 months, 4.6% (95% CI, 3.1%-6.6%) at 6 months, and 6.3% (95% CI, 4.6%-8.7%) at 1 year (Figure 2 and eTable 4 in Supplement 2). At 6 months, risk ranged from 2.3% to 9.3%, depending on surgery type.

The cumulative incidence of recurrence in patients unexposed to surgery was 0.8% (95% CI, 0.6%-1.1%) at 3 months. The landmark analysis yielded similar results. Recurrence risk was highest within the first month of surgery (HR, 6.8; 95% CI, 3.9-11.9) and remained increased up to approximately 6 months after surgery (HR, 1.7; 95% CI, 0.8-3.7) (Table 2; eTable 5 in Supplement 2).

**Table 2.** Association of Surgery With Recurrence Risk in Patients With a History of VTE per Time Window^a^

VTE Recurrence Risk^b^	No. of Operations	Person-Years	No. of VTEs	Adjusted HR (95% CI)^c^			
				<1 mo	1-<3 mo	3-<6 mo	6-12 mo
Patients without surgery^d^	0	18 636	571	NA	NA	NA	NA
Total surgical procedures	580	263	30	6.8 (3.9-11.9)	2.5 (1.2-5.1)	1.7 (0.8-3.7)	1.3 (0.7-2.6)
Restriction analysis 1^e^	533	240	29	6.5 (3.5-11.8)	2.8 (1.4-5.6)	1.9 (0.9-4.1)	1.5 (0.8-2.9)
Restriction analysis 2^f^	478	219	24	5.8 (3.0-11.2)	1.9 (0.8-4.5)	2.1 (1.0-4.4)	1.6 (0.8-3.2)
Total orthopedic surgical procedures	219	101	11	4.0 (1.3-12.4)	3.3 (1.2-8.8)	2.7 (1.0-7.3)	1.6 (0.6-4.3)
Restriction analysis 1^e^	207	95	11	4.2 (1.4-13.2)	3.4 (1.3-9.2)	2.9 (1.1-7.7)	1.7 (0.6-4.6)
Restriction analysis 2^f^	200	92	11	4.3 (1.4-13.4)	3.5 (1.3-9.3)	2.9 (1.1-7.8)	1.8 (0.7-4.7)
Total nonorthopedic surgical procedures	403	181	19	8.2 (4.4-15.3)	1.9 (0.7-5.1)	1.9 (0.8-4.7)	1.0 (0.4-2.6)
Restriction analysis 1^e^	368	165	18	7.4 (3.7-14.9)	2.2 (0.8-5.8)	2.2 (0.9-5.3)	1.1 (0.4-2.9)
Restriction analysis 2^f^	317	145	13	6.4 (2.8-14.3)	0.6 (0.1-4.3)	2.5 (1.0-5.9)	1.2 (0.5-3.3)

Abbreviations: HR, hazard ratio; NA, not applicable; VTE, venous thromboembolism.

^a^ Separate time windows exclude previous risk periods.

^b^ For first surgery during follow-up period, which began on stop date of anticoagulation therapy after first VTE.

^c^ Hazard ratio adjusted for age and sex.

^d^ Person-years and number of VTEs only shown for the complete period of increased risk (0-6 months). Numbers (operations, person-years, and VTEs) do not sum to the total because the first surgery can represent all surgery types, depending on the risk group of interest.

^e^ Excluding patients with cancer diagnoses within 5 years before and 6 months after first VTE.

^f^ Excluding patients with cancer diagnoses within 5 years before and 6 months after first VTE and who developed cancer during follow-up.

Vascular and outpatient surgical procedures were associated with the lowest recurrence risk at 6 months (vascular: HR, 2.3; 95% CI, 0.6-8.8; outpatient: HR, 3.1; 95% CI, 1.4-6.7). Patients who had undergone gastrointestinal procedures (eg, esophagus, stomach, bowel, or rectal operations) had a high risk of recurrent VTE at 6 months (HR, 8.4; 95% CI, 4.0-17.8) (Figure 2).

Nonorthopedic surgical procedures were associated with a higher risk of recurrence at 1 month (HR, 8.2; 95% CI, 4.4-15.3) compared with orthopedic surgery (HR, 4.0; 95% CI, 4.4-15.3) (Table 2). Furthermore, patients who had undergone major surgery had a higher risk of recurrent VTE than those who had undergone minor surgery.

### Cancer-Related Surgery

During follow-up, 110 patients developed cancer (eFigure in Supplement 2), of whom 55 underwent surgical procedures within our period of interest (44 first operations during follow-up and 11 second or third operations). The most commonly performed operations were related to breast cancer (n = 12), colon cancer (n = 7), or rectal cancer (n = 8). The absolute recurrence risk at 6 months after cancer-related surgery was 9.3% (95% CI, 3.6%-22.9%) (Figure 2).

The risk of recurrent VTE in all patients who underwent surgery did not change when we excluded patients with a cancer diagnosis within 5 years before (or within 6 months following) their first VTE. Subsequent exclusion of patients who developed cancer during follow-up resulted in somewhat lower risks, most strongly pronounced in patients who underwent nonorthopedic surgery (patients who underwent nonorthopedic surgery: risk at 6 months, 4.0; 95% CI, 2.5-6.5; after exclusion of patients who developed cancer during follow-up: risk at 6 months, 3.2; 95% CI, 1.7 to 5.9; difference, −0.8%) (eTable 6 in Supplement 2).

### Factors Associated With Recurrence

Factors associated with increased risk of VTE recurrence in patients who underwent surgery included factor V Leiden mutation (HR, 3.4; 95% CI, 1.6-7.4) and male sex (HR, 2.7; 95% CI, 1.3-5.8) (Table 3). Men with factor V Leiden who underwent surgery had an 8.5-fold increased risk of VTE recurrence compared with women without factor V Leiden who underwent surgery (cumulative incidence at 6 months: 18.0%; 95% CI, 9.0%-34.1%). For those with a first unprovoked VTE, recurrence risk was 6.7% (95% CI, 3.6%-12.6%), while patients with a first provoked VTE also had a high risk of recurrence (HR, 5.1; 95% CI, 3.4-7.7) at 6 months. For patients who underwent surgery 2 or more years after their first VTE, absolute recurrence risks were slightly lower in most risk groups (Table 3).

**Table 3.** Factors Associated With Recurrent VTE Parallel to Surgery

Risk Factor	Person-Years (n = 263)	No. of VTEs (n = 30)	All Patients Within 6 mo of Surgery, Adjusted HR (95% CI)^a^	Cumulative Incidence at 6 mo, % (95% CI)	
				All Patients Who Underwent Surgery	Patients Who Underwent Surgery >2 y After First VTE
Sex					
Women	153	10	1 [Reference]	3.1 (1.7-5.7)	1.8 (0.6-5.5)
Men	109	20	2.7 (1.3-5.8)	8.5 (5.6-12.8)	6.3 (2.4-12.7)
Age	NA	NA	1.0 (1.0-1.1)	NA	NA
Blood type					
O	75	7	1 [Reference]	4.3 (2.1-8.9)	1.3 (0.2-8.5)
XO	127	16	1.3 (0.5-3.2)	6.0 (3.7-9.5)	6.0 (3.1-11.7)
Non-O	39	5	1.3 (0.4-4.3)	6.3 (2.7-14.5)	NA^b^
FVL mutation					
Absent	207	18	1 [Reference]	4.2 (2.6-6.5)	2.3 (1.0-5.5)
Present	35	10	3.4 (1.6-7.4)	12.8 (7.1-22.5)	10.7 (4.2-26.0)
Prothrombin 20210A mutation					
Absent	231	27	1 [Reference]	5.5 (3.8-7.9)	3.7 (2.0-7.0)
Present	10	1	0.8 (0.2-4.5)	5.0 (0.7-30.5)	NA^b^
First VTE					
DVT	183	20	1 [Reference]	5.2 (3.4-7.9)	2.6 (1.1-6.2)
PE (with or without DVT)	79	10	1.2 (0.5-2.5)	5.9 (3.2-10.7)	5.9 (2.5-13.5)
Obesity					
No	88	9	1 [Reference]	5.0 (2.6-9.4)	2.2 (0.6-8.5)
Yes^c^	174	21	1.2 (0.5-2.5)	5.6 (3.4-8.5)	4.4 (2.2-8.6)
Comorbidity					
No	184	20	1 [Reference]	5.1 (3.4-7.9)	3.1 (1.4-6.7)
Yes^d^	46	3	0.6 (0.2-2.1)	3.1 (1.0-9.3)	2.2 (0.3-14.5)
First VTE provoked					
No	62	9	1 [Reference]	6.7 (3.6-12.6)	4.2 (1.4-12.4)
Yes^e^	195	21	0.7 (0.3-1.6)	5.1 (3.4-7.7)	3.6 (1.7-7.3)
Risk score: sex and/or FVL mutation^f^					
0 (Female and no FVL mutation)	124	6	1 [Reference]	2.3 (1.1-5.1)	1.5 (0.4-5.8)
1 (Female and FVL mutation)	18	3	3.5 (0.9-14.2)	7.6 (2.5-21.9)	5.3 (0.8-31.9)
2 (Male and no FVL mutation)	83	12	2.8 (1.1-7.6)	6.8 (3.9-11.7)	3.7 (1.2-10.9)
3 (Male and FVL mutation)	17	7	8.5 (2.8-25.2)	18.0 (9.0-34.1)	15.8 (5.4-41.4)
Time to surgery, y					
<1	84	12	1 [Reference]	6.8 (3.9-11.7)	NA
1-2	51	8	1.1 (0.5-2.7)	7.1 (3.6-13.8)	NA
>2	127	10	0.6 (0.2-1.3)	3.7 (2.0-6.8)	3.7 (2.0-6.8)

Abbreviations: DVT, deep vein thrombosis; FVL, factor V Leiden; HR, hazard ratio; NA, not applicable; PE, pulmonary embolism; VTE, venous thromboembolism.

^a^ Adjusted for time to surgery.

^b^ Not estimable because no events occurred within this subgroup.

^c^ Obesity defined as body mass index greater than 25 (calculated as weight in kilograms divided by height in meters squared).

^d^ Comorbidity denotes presence of any major illness, as listed in Table 1.

^e^ Provoked first VTE defined as provoked by either cancer, surgery, immobilization, travel, pregnancy, or hormone use.

^f^ Hazard ratios shown for patients with available information on FVL mutation.

## Discussion

### Principal Findings

This study demonstrated that patients with VTE who underwent subsequent surgery had a high risk of developing recurrent VTE up to 6 months after surgery, with an overall risk of 4.6% (range, 2.3%-9.3%, depending on surgery type). Cancer-related surgery, major orthopedic, gastrointestinal, and heart-lung procedures were associated with the highest risks of recurrence, while the risks of outpatient and minor surgery were increased to a lesser extent. In addition, we showed that men and patients with factor V Leiden had a higher risk of developing recurrent VTE.

### Comparison With Previous Studies

In 2015, a population-based case-cohort study^6^ showed that patients who underwent surgery for which they were also hospitalized after their first VTE had a 6-fold increased risk of developing in-hospital recurrent VTE compared with patients with a history of VTE without surgery (HR, 5.9; 95% CI, 3.3-10.4).^6^ This relative risk declined to 1.9 (95% CI, 1.1-3.2) within 3 months. While our results are generally in line with this study, we were able to estimate risks for different types of surgery, which showed substantial variation. In 2010, Bahl et al^4^ performed a large cohort study in which 8216 patients who underwent general, vascular, and urologic surgery (excluding outpatient surgery) were retrospectively analyzed for the occurrence of VTE. In that study, 285 patients with a history of VTE (4.2%) developed recurrence within 30 days of surgery.^4^ Our study showed similar rates for gastrointestinal and cancer-related surgery, but the 30-day risk of recurrence in all surgical patients was lower, at 2.1%. The study by Bahl et al^4^ collected data from medical records, which could have led to an underestimation of the number of patients with a history of VTE, hence leading to a higher absolute risk. It is well known that these registry studies have implicit drawbacks, such as misclassification, which tend to underestimate absolute risks.

### Clinical Implications and Future Research Perspective

To our knowledge, this is the first study that gives detailed information on absolute recurrence risks of VTE following various types of surgery in combination with patient characteristics. Our results indicate that there is much heterogeneity in risk dependent on these factors. Given that VTE is the most preventable death in hospitals, and 60% of VTE cases occur during or following hospitalization,^18^ it is important to acknowledge the high recurrence risks associated with surgery when a patient has a history of VTE.

Contemporary guidelines for surgical patients advise clinicians to provide thromboprophylaxis therapy after most procedures, although the treatment duration is debated. Frequently, a distinction is made between high- and low-risk surgical patients, based on the procedure itself and a patient’s comorbidities.^1,9^ A history of VTE will almost always warrant thromboprophylactic therapy after any surgical intervention. For instance, according to the American College of Chest Physicians guideline on thromboprophylaxis in nonorthopedic surgical patients, virtually all patients with a history of VTE who undergo surgery are to be treated with thromboprophylaxis unless contraindicated (eg, high bleeding risk). Only young patients with a history of VTE who undergo minor surgery can be withheld from prophylactic therapy.^1^ Similarly, in the UK guidelines on prevention of VTE (National Institute for Health and Care Excellence), it is advised to offer VTE prophylaxis for 5 to 7 days for all patients undergoing gastrointestinal, gynecological, thoracic, or urologic surgery who are at increased risk (which includes patients with a history of VTE).^9^

Despite these recommendations, 4.6% of patients undergoing surgery developed a recurrent event within 6 months in our study. It is therefore highly doubtful that the current practice is sufficiently effective for recurrence prevention. Interestingly, the 1-month risk following nonorthopedic surgery was higher than following orthopedic surgery (2.3% vs 1.4%), which may reflect different thromboprophylaxis strategies between these groups (ie, a more aggressive and longer duration of prophylaxis following orthopedic surgery).^1,2^ However, risk differences between these groups evened out after 6 months, since the risk in the orthopedic group remained high. Our finding that the recurrence risk remained increased up to 6 months after the surgical intervention supports a policy with extended duration of thromboprophylactic therapy, not restricted to in-hospital prophylaxis. This should be tested in further trials. Furthermore, our study indicates that some patients are at additional high risk; for instance, the size of the risk is associated with the type of surgery as well as on patient characteristics, such as male sex and factor V Leiden mutation. Moreover, 7 of 39 men with factor V Leiden mutation (18%) developed recurrence within 6 months after surgery. Furthermore, we showed that patients who underwent surgery more than 2 years after their first VTE had a slightly lower (but still increased) risk of recurrence. Hence, high-risk patients—those who undergo major surgical procedures or those who have multiple risk factors and are undergoing low-risk procedures—may need prolonged anticoagulation therapy (or a higher dosage) following surgery to prevent recurrence. However, such advice should be carefully weighed against individual bleeding risks and warrants additional studies.

### Strengths and Limitations

The main strengths of our study are the time-dependent analysis in a large unselected sample of patients who underwent surgery (largest to date, to our knowledge), long follow-up period, and the objective classification of surgery. By handling surgery as a time-dependent covariate in our model, we could adjust for time to surgery from start of follow-up. In addition, patients contributed to both exposure and nonexposure time during follow-up, so all patients also functioned as their own control. Furthermore, the large sample size led to precise estimations of the actual recurrence risk of VTE, and the objective classification of surgery led to the elimination of recall bias.

Our study had limitations. One limitation of our study is that we did not have information on thromboprophylaxis therapy following the surgical intervention. However, a nationwide survey study among all surgical departments in the Netherlands^19^ performed within the same time frame as our study showed that adherence to antithrombotic guidelines in surgical patients was 92%. (Dutch guidelines were comparable with the American College of Chest Physicians guidelines at the time of study.) Because all guidelines advise clinicians to provide thromboprophylaxis during hospitalization to high-risk surgical patients (ie, patients with a history of VTE), it is highly unlikely that patients did not receive thromboprophylaxis after surgery. Furthermore, according to the survey, 76% of all surgeons took additional antithrombotic measures into consideration (such as double-dose prophylactic therapy) when patients had obesity, a personal history of VTE, or older age. Still, some patients undergoing minor surgery might have been withheld thromboprophylaxis or could have decided not to use it. Although we are confident that thromboprophylaxis was applied according to the guidelines for most, it might be worthwhile to consider 2 extreme situations to assess the effect of complete use or complete nonuse of prophylaxis on our results. On the one hand, suppose that no single patient in our study who underwent surgery received thromboprophylactic therapy. Then, assuming a risk reduction of 50% by using thromboprophylaxis, the cumulative incidence at 6 months would still be high even if patients had received thromboprophylaxis, ie, 2.3% following any surgery (4.6% × 0.5; ie, half the 6-month incidence rate we found). On the other hand, assuming that doctors had fully complied with antithrombotic guidelines, which is most likely, the cumulative incidence following any surgery with thromboprophylaxis is 4.6% (as presented in this study), and it would have been 9.2% (ie, 4.6% × 2) if no patients had received thromboprophylaxis. Therefore, it is clear that, at any rate of prophylaxis, patients with a history of VTE who undergo surgery have a high risk of developing a new thrombotic event, ie, within a range of 2.3% to 9.2%, depending on the type of surgery. This suggests that current thromboprophylactic measures for patients with a history of VTE are not sufficiently effective.

As a possible second limitation, we only adjusted for age and sex in the Cox regression. Of note, our primary goal was to show the absolute risk of VTE following surgery and not to show whether surgery is a causal (provoking) risk factor. The former aim has clinical meaning, whereas the causal role of surgery in VTE has been known for decades. Third, patients 70 years and younger were included in the MEGA study; thus, the generalizability of our study is limited to individuals in that age range. However, it is not to be expected that the conclusions of this study would be different for older patients. Also, the highest-risk patients (ie, those receiving long-term anticoagulation therapy following their first VTE) were excluded from the analyses.

## Conclusions

This study found that patients with a history of VTE who underwent surgery had a high recurrence risk of VTE, which remained increased up to 6 months after surgery. High-risk individuals may be identified based on the type of surgery and the presence of additional factors. Our results stress the need for a revision of the thromboprophylactic approach following surgery in patients with a history of VTE, the duration and dosage of which may need to be intensified and individualized.

## Notes

Supplement 1. **Trial Protocol**

Supplement 2. **eMethods. **Classification of Recurrence **eFigure. **Study Flowchart **eTable 1. **Sensitivity Analysis of Association of Surgery With Recurrence Risk in Patients With a History of VTE Without Censoring of Second Surgery **eTable 2. **Overview and Number of All Surgical Procedures During Follow-up **eTable 3. **Detailed List of All Surgical Procedures During Follow-up **eTable 4. **Absolute Risk of Recurrent Venous Thromboembolism After Surgery in Patients With a History of VTE **eTable 5. **Association of Surgery With Recurrence Risk in Patients With a History of VTE **eTable 6. **Restriction Analysis of Absolute Risk of Recurrent Venous Thromboembolism After Surgery in Patients With a History of VTE, Excluding Patients With Cancer
