# Typographical Error in Results

**DOI:** 10.1001/jamanetworkopen.2019.6420

**Published:** 2019-06-07

In the Original Investigation titled “Risk and Risk Factors Associated With Recurrent Venous Thromboembolism Following Surgery in Patients With History of Venous Thromboembolism,”^1^ published May 10, 2019, there was a typographical error in the VTE Recurrence Risk subsection of the Results section. The first sentence of the final paragraph should read as follows: “Nonorthopedic surgical procedures were associated with a higher risk of recurrence at 1 month (HR, 8.2; 95% CI, 4.4-15.3) compared with orthopedic surgery (HR, 4.0; 95% CI, 1.3-12.4) (Table 2).” This article has been corrected.^1^
