# Supplementary material for: Risk and Risk Factors Associated With Recurrent Venous Thromboembolism Following Surgery in Patients With History of Venous Thromboembolism
Source: JAMA Netw Open. 2019 May 10;2(5):e193690. doi: 10.1001/jamanetworkopen.2019.3690 (PMC6512304; doi:10.1001/jamanetworkopen.2019.3690)
Supplement: Supplement 1. — Trial Protocol [file jamanetwopen-2-e193690-s001.pdf]

Study protocol

Proposal number: WC-KE/ys/2016-A037

Title/subject : The risk of recurrent venous thrombosis in patients with a history of venous thrombosis undergoing surgery

Key words: surgery, venous thrombosis, recurrence, risk, hospital admission
Date: 19-11-2015

Researcher(s): B. Nemeth, W.M. Lijfering, S.C. Cannegieter

Please send this proposal to the secretary of the Scientific Committee of the Department of Clinical Epidemiology.

Background+ relevance (max ½ A4, shorter preferred)

a. Hypothesis

Patients can receive prolonged anticoagulant therapy after a first venous thrombotic event to prevent recurrence, although at the cost of bleeding. Therefore it is crucial to estimate an individual's risk for recurrence, to personalize anticoagulant therapy and minimize bleeding risk. Surgery is a major risk factor for the development of venous thrombosis (VT).[1] For this reason, routine thromboprophylaxis is strongly recommended around major surgery.[1;2] Although the risk of a first VT after surgery is studied extensively, there is less information about the risk to develop a recurrent VT in patients with a history of VT who need to undergo surgery. Some studies report a high risk of recurrence; for example, one study reported that despite mechanical and pharmacological anticoagulant treatment with warfarin, patients with a history of VT who underwent a lower limb joint replacement had a relative risk of 6.3 (95%CI 2.3 to 17.5) at 90 days for recurrent VT compared with individuals that had no history of VT.[3] Another study in patients undergoing colorectal surgery (for any cause) reported that patients with a history of VT had a 7.6-fold increased risk for recurrence (95%CI 5.1 to 11.32) compared to those without a history of VT, despite prophylactic therapy.[4] As far we know, only one study addressed whether patients with previous venous thrombosis are at increased risk of a recurrent event, while undergoing surgery compared with such patients who do not have surgery .[5] In that study authors observed that patients with VT who underwent surgery had a 3-10 fold increased risk of a recurrent event up to 92 days post dismissal as compared with patients with VT who did not undergo surgery over a same follow-up time. However, authors were not able to distinguish those who received thromboprophylaxis from those who did not receive thromboprophylaxis and could also not distinguish between various types of surgery.

Since many patients with a history of VT undergo surgery, and the scarcity of studies that have been performed on this issue, we decided to research the risk of recurrence in these patients to guide physicians on prescribing anticoagulant medication.

b. Objective

1. To estimate the absolute risk of recurrent VT in patients with a history of VT who undergo various types of surgery.

2. To identify risk factors for recurrent VT in these patients.

2. Study design

a. Population description (inclusion/exclusion criteria)

For this study we will use data from the MEGA follow-up study. 4731 patients with a first venous thrombosis were followed over time for recurrent venous thrombosis until 2008-2009. Information on the occurrence of a recurrent event was collected by means of a short questionnaire or patients were contacted by phone. Detailed information about the recurrent event was retrieved from questionnaires, anticoagulation clinics, treating physicians or cause of death statistics. At the end of study about 700 cases with a recurrent venous thrombosis were identified.

Participants completed a questionnaire on putative risk factors for recurrent venous thrombosis (including [type of] surgery). Despite this information, we will not use this data as information bias (recall bias) is likely to be present. The MEGA follow-up database has been linked to the Central Bureau of Statistics (CBS) database of the Netherlands with information on all surgical procedures (including dates) for all individuals that participated in the study. We will use these data to identify patients who underwent a surgical procedure during the period of interest.

b. Exposure (independent variable)

We are interested in all surgical procedures (i.e. orthopedic, non-orthopedic, gynecological, urological, abdominal, vascular, neurosurgical, etc), both daycare procedures and operations whereby patients are hospitalized for one or more days (see table under 4b), and we will differentiate between major and non-major surgery. These procedures are identified using ICD-coding that is registered in the CBS database.

c. Outcome (dependent variable)

The outcome of interest is recurrent VT. In the MEGA follow-up study, events are identified as certain and uncertain recurrences. For our study we will consider certain recurrences only and censor follow-up time in case a non-certain recurrent event has occurred. In addition, uncertain cases will be included as recurrent events in a sensitivity analysis.[6]

d. Confounding factors and selection bias

Confounding

Lots of acquired risk factors can confound the association between surgery and recurrent venous thrombosis. Patients in the MEGA study completed a questionnaire on putative risk factors for a first VT, also, later in the follow-up study, about half of the participants completed a second questionnaire on risk factors for a recurrent VT. We will adjust for baseline factors including age, sex,

body mass index (BMI), and chronic diseases such as liver and kidney failure and inflammatory diseases (including rheumatoid arthritis) (full definition of chronic and inflammatory disease in MEGA are provided in Ocak et al. JTH 2013 and Nemeth et al. PLOS Med 2015). We will impute data when data on one of these confounders are missing. Patients with cancer (at baseline and during follow-up) will be excluded from all analyses.

There are no data available on thromboprophylactic therapy during or after surgery. All guidelines advise to give prophylactic (low dose) anticoagulant therapy during surgery for patients with a history of VT. Therefore we assume that this has been the case for all patients who underwent surgery.

Selection bias

We chose to collect surgery data from the Central Bureau of Statistics of the Netherlands to avoid selection bias induced by the fact that not all participants completed the questionnaire on putative risk factors (such as surgery) for a recurrent event. 95% of all patients are linked to CBS data, individuals who are not linked will be excluded from the analysis, an event that is considered to be random.[7]

3. Statistical analysis

a. Methods

Follow-up time will be calculated from the start of follow-up (i.e. 1. from stop date anticoagulant treatment after first VT and 2. from date of first VT onwards as a sensitivity analysis) until the end of follow-up (end of study, dead, recurrent event or loss to follow-up, whichever occurs first). The time window of surgery exposure for each individual is defined as 3-months from the surgery date. The total follow-up time in which patients are not exposed will be calculated as the total follow-up time minus the exposure time. As a sensitivity analysis, we will vary the time window of exposure because it is not known for how long the VT risk is increased after surgery. Therefore we will also consider a 1-month, 6-month and 1-year time window after surgery. We will calculate cumulative incidences for recurrent venous thrombosis for the exposed time windows and incidence rates by dividing the number of events by person-time for the exposed and the unexposed time periods.

Cox-proportional hazards modeling (with a time-dependent co-variate [surgery]) will be used to calculate a hazard ratio for developing recurrence. Also, we will estimate Hazard Ratios for several types of surgery on recurrence risk, and adjust for important confounders. Absolute risks for

recurrent VT will be calculated for several types of surgery (for all different time windows after exposure).

To identify high risk groups, we will perform a matched case-control study within this cohort. Cases are patients with a recurrent event who underwent surgery, controls are patients without a recurrent event who also underwent surgery. Cases and controls will be matched for [type of] surgery exposure. Four possible risk factors will be tested: 1. The presence of Factor V Leiden or non-O blood type or prothrombin mutation, 2. provoked or unprovoked first event, 3. DVT or PE as a first event and 4. sex. Odds ratios with 95% CIs will be calculated for these risk factors using logistic regression analysis. A prediction model will be developed if the number of cases is sufficient enough.

b. Sample size considerations

4731 patients were followed over time of which 700 developed a recurrence. Because we have no information on the frequency of the exposure, sample size calculations are estimations. If we assume an exposure to surgery of 10% and a 5% recurrent VT rate in the unexposed, we are able to find a relative risk of 1.6, with a power of 80%.

4. Tables

a. General characteristics table

**General characteristics**				
Study population				
Age, mean (sd)				
Sex, male (%)				
BMI, mean (sd)				
Comorbidity				
Major illness				
No				
Yes	591			
	Liver disease		27	
	Kidney disease		60	
	Rheumatoid arthritis			145
	Multiple sclerosis		30	
	Heart failure		76	
	Hemorrhagic stroke			36
	Arterial thrombosis			299
		MI	137	
		Angina	64	
		Ischemic stroke		41
		TIA	61	
		PVD	55	
Inflammatory disease				

Surgical procedures	
Surgery (all)	
Orthopaedic surgery (%)	
Total knee replacement	
Total hip replacement	
Hip fracture surgery	
Knee arthroscopy	
Lower limb / foot surgery	
Other	
Non-orthopaedic surgery (%)	
Gynaecological	
Urological	
Neurosurgical	
Abdominal	
Minor abdominal	
Major abdominal	
Vascular	
Minor vascular	
Major vascular	

b. **Planned table(s)**

**Cumulative Incidence VT recurrent risk**	**number of patients**	**1-month** CI % (95%CI)	**3-months** CI % (95%CI)	**6-months** CI % (95%CI)	**1-year** CI % (95%CI)
**Surgery (all),**					
**Orthopaedic surgery (all).**					
Major orthopaedic surgery					
Total knee replacement					
Total hip replacement					
Hip fracture surgery					
Non-major orthopaedic surgery					
Knee arthroscopy					
Lower limb / foot surgery					
Other					
**Non-orthopaedic surgery (all)**					
Gynaecological					
Urological					
Neurosurgical					
Abdominal					
Minor abdominal					
Major abdominal					
Vascular					
Minor vascular					
Major vascular					
**Non-orthopaedic day-care surgery**					

**VT recurrence risk**	**number of patients**	**1-month** HR (95%CI)	**3-month** HR_{adj}* (95%CI)	**6-months** HR (95%CI)	**1-year** HR_{adj}* (95%CI)
**No surgery**		ref	ref	ref	ref
**Surgery (all)**					
**Orthopaedic surgery (all)**					
Major orthopaedic surgery					
Total knee replacement					
Total hip replacement					
Hip fracture surgery					
Non-major orthopaedic surgery					
Knee arthroscopy					
Lower limb / foot surgery					
Other					
**Non-orthopaedic surgery (all)**					
Gynaecological					
Urological					
Neurosurgical					
Abdominal					
Minor abdominal					
Major abdominal					
Vascular					
Minor vascular					
Major vascular					
**Non-orthopaedic day-care surgery**					

* Adjusted for age, sex, BMI and all comorbidities

c. Planned figure(s)

None

5. **Reference list**

Reference List

1. Gould MK, Garcia DA, Wren SM et al. Prevention of VTE in nonorthopedic surgical patients: Antithrombotic Therapy and Prevention of Thrombosis, 9th ed: American College of Chest Physicians Evidence-Based Clinical Practice Guidelines. Chest 2012;141:e227S-e277S.

2. Falck-Ytter Y, Francis CW, Johanson NA et al. Prevention of VTE in orthopedic surgery patients: Antithrombotic Therapy and Prevention of Thrombosis, 9th ed: American College of Chest Physicians Evidence-Based Clinical Practice Guidelines. Chest 2012;141:e278S-e325S.

3. Allen D, Sale G. Lower limb joint replacement in patients with a history of venous thromboembolism. Bone Joint J. 2014;96-B:1515-1519.

4. Nelson DW, Simianu VV, Bastawrous AL et al. Thromboembolic Complications and Prophylaxis Patterns in Colorectal Surgery. JAMA Surg. 2015;150:712-720.

5. Heit JA, Lahr BD, Ashrani AA, Petterson TM, Bailey KR. Predictors of venous thromboembolism recurrence, adjusted for treatments and interim exposures: a population-based case-cohort study. Thromb.Res. 2015;136:298-307.

6. Timp JF, Lijfering WM, Flinterman LE et al. Predictive value of factor VIII levels for recurrent venous thrombosis: results from the MEGA follow-up study. J.Thromb.Haemost. 2015

7. Roach RE, Lijfering WM, Flinterman LE, Rosendaal FR, Cannegieter SC. Increased risk of CVD after VT is determined by common etiologic factors. Blood 2013;121:4948-4954.
