# Supplementary material for: Risk and Risk Factors Associated With Recurrent Venous Thromboembolism Following Surgery in Patients With History of Venous Thromboembolism
Source: JAMA Netw Open. 2019 May 10;2(5):e193690. doi: 10.1001/jamanetworkopen.2019.3690 (PMC6512304; doi:10.1001/jamanetworkopen.2019.3690)
Supplement: Supplement 2. — eMethods. Classification of Recurrence eFigure. Study Flowchart eTable 1. Sensitivity Analysis of Association of Surgery With Recurrence Risk in Patients With a History of VTE Without Censoring of Second Surgery eTable 2. Overview and Number of All Surgical Procedures During Follow-up eTable 3. Detailed List of All Surgical Procedures During Follow-up eTable 4. Absolute Risk of Recurrent Venous Thromboembolism After Surgery in Patients With a History of VTE eTable 5. Association of Surgery With Recurrence Risk in Patients With a History of VTE eTable 6. Restriction Analysis of Absolute Risk of Recurrent Venous Thromboembolism After Surgery in Patients With a History of VTE, Excluding Patients With Cancer [file jamanetwopen-2-e193690-s002.pdf]

Supplementary Online Content

Nemeth B, Lijfering WM, Nelissen RGHH, et al. Risk and risk factors associated with recurrent venous thromboembolism following surgery in patients with history of venous thromboembolism. *JAMA Netw Open.* 2019;2(5):e193690. doi:10.1001/jamanetworkopen.2019.3690

eMethods. Classification of Recurrence

eFigure. Study Flowchart

eTable 1. Sensitivity Analysis of Association of Surgery With Recurrence Risk in Patients With a History of VTE Without Censoring of Second Surgery

eTable 2. Overview and Number of All Surgical Procedures During Follow-up

eTable 3. Detailed List of All Surgical Procedures During Follow-up

eTable 4. Absolute Risk of Recurrent Venous Thromboembolism After Surgery in Patients With a History of VTE

eTable 5. Association of Surgery With Recurrence Risk in Patients With a History of VTE

eTable 6. Restriction Analysis of Absolute Risk of Recurrent Venous Thromboembolism After Surgery in Patients With a History of VTE, Excluding Patients With Cancer

This supplementary material has been provided by the authors to give readers additional information about their work.

© 2019 Nemeth B et al. *JAMA Network Open.*

eMethods. Classification of Recurrence

To be classified as a certain recurrence, a reported recurrence should fulfil one of the following criteria.

1. A discharge letter was present concluding a diagnosis of recurrence, based on available clinical and radiological data. This recurrence should be in a different vein or in a different part of the body than the first event. The discharge letter had to contain information about instrumental diagnostic procedures. If location of either first or second thrombosis was not known or was similar to the first event, an event was still classified as certain if at least three months had passed since the first thrombosis.
2. A discharge letter was not available (e.g. when treating physician was unknown) but both the anticoagulation clinic and the patient reported a recurrence at a clearly different location than the first event (contralateral leg, DVT after PE or vice versa) or a time period of more than a year had passed between the two events.
3. A registered cause of death from PE or DVT at least six months after the first event.

Uncertain recurrences were defined by four criteria, one of which had to apply:

1. A diagnosis of a possible recurrence in the discharge letter, where clinical and radiological data could not distinguish between an extension of the first and a new thrombotic event.
2. A discharge letter was not available but both the patient and the anticoagulation clinic reported a recurrence within a year after the first event.
3. Information was only available from either the patient or the anticoagulation clinic.
4. A registered cause of death from PE or DVT within six months after the first event.

© 2019 Nemeth B et al. *JAMA Network Open.*

eFigure 1: Study Flowchart

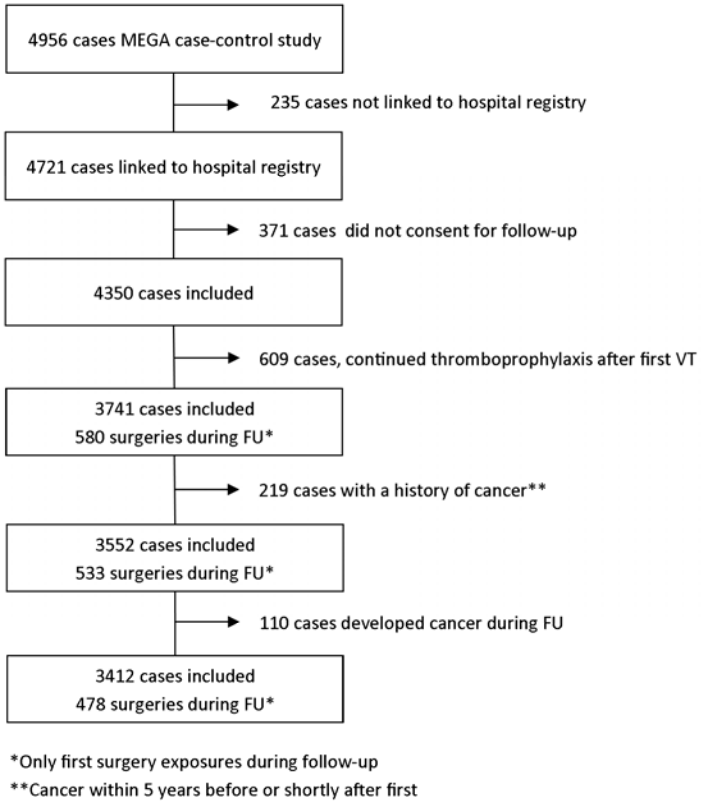

FU denotes follow-up, VT denotes Venous Thrombosis
*Only first surgery exposures during follow-up
**Cancer within 5 years before or shortly after first VTE (analysis shown in table 3b).

© 2019 Nemeth B et al. *JAMA Network Open.*

eTable 1. Sensitivity Analysis of Association of Surgery With Recurrence Risk in Patients With a History of VTE Without Censoring of Second Surgery

**VTE recurrence risk****	**0 to 1-month** HR_{adj}* (95%CI)	**0 to 3-months** HR_{adj}* (95%CI)	**0 to 6-months** HR_{adj}* (95%CI)	**0 to 1-year** HR_{adj}* (95%CI)
**No surgery (ref)**	ref	ref	ref	ref
**Surgery (all)**	6.4 (3.6 to 11.3)	3.8 (2.4 to 6.0)	2.9 (2.0 to 4.3)	2.3 (1.6 to 3.2)
Major surgery	6.7 (3.5 to 13.0)	3.8 (2.2 to 6.5)	2.9 (1.8 to 4.6)	2.4 (1.6 to 3.6)
Minor surgery	4.6 (1.5 to 14.2)	2.9 (1.2 to 7.0)	2.3 (1.1 to 4.8)	1.8 (1.0 to 3.4)
Outpatient surgery	4.8 (1.5 to 14.8)	2.4 (0.9 to 6.4)	2.0 (0.9 to 4.4)	1.3 (0.6 to 2.7)
Cancer surgery	8.4 (2.1 to 33.8)	3.9 (1.0 to 15.6)	3.3 (1.0 to 10.1)	2.1 (0.7 to 6.4)
**Orthopaedic surgery (all)**	4.0 (1.3 to 12.5)	3.0 (1.4 to 6.8)	2.9 (1.5 to 5.4)	2.5 (1.5 to 4.2)
Major orthopaedic	6.4 (1.6 to 25.6)	3.5 (1.1 to 10.7)	3.2 (1.3 to 7.8)	3.2 (1.3 to 7.8)
Minor orthopaedic	2.1 (0.3 to 15.2)	2.5 (0.8 to 7.8)	2.4 (1.0 to 5.7)	1.9 (0.9 to 3.9)

**Non-orthopaedic surgery**	7.4 (3.8 to 14.3)	3.9 (2.2 to 6.8)	2.7 (1.6 to 4.4)	1.9 (1.2 to 3.0)
Abdominal surgery	5.0 (1.2 to 20.0)	2.7 (0.9 to 8.3)	2.5 (1.0 to 6.0)	2.0 (0.9 to 4.1)
Gastrointestinal surgery	12.3 (3.9 to 38.4)	7.2 (3.0 to 17.4)	4.9 (2.2 to 11.1)	3.4 (1.6 to 7.2)
Heart Lung surgery	8.2 (1.1 to 58.3)	5.9 (1.5 to 23.9)	3.1 (0.8 to 12.5)	1.8 (0.4 to 7.1)
Vascular surgery	7.4 (1.9 to 29.9)	2.6 (0.6 to 10.4)	1.4 (0.4 to 5.7)	0.8 (0.2 to 3.2)
Gynaecological surgery	Not estimable	Not estimable	Not estimable	0.8 (0.1 to 5.5)

*It is important to note that for example 0 to 1-year period also includes the (high) hazard of the first period following surgery.

¶Only for first surgery exposure during follow-up, start follow-up is stop-date of anticoagulant therapy after first VTE.

±Hazard Ratio (HR_{adj}) adjusted for age & sex.

© 2019 Nemeth B et al. *JAMA Network Open.*

eTable 2. Overview and Number of All Surgical Procedures During Follow-up

**Surgical procedures**	**1st surgery*** number	**All surgeries** number
**Surgery (all)**	580	808
Major surgery	441	578
Minor surgery	198	230
Outpatient surgery	201	238
Cancer related surgery	44	55
**Orthopaedic surgery (%)**	219	275
Major orthopaedic surgery	101	125
Minor orthopaedic surgery	135	150
**Non-orthopaedic surgery (%)**	403	533†
Vascular surgery	89	109
Gastrointestinal surgery	73	89
Abdominal surgery	120	133
Heart lung surgery	89	52
Gynaecological surgery	73	80

*Numbers do not add up because the first operation can be both, for example, major or minor surgery (or because some surgeries were of other nature).

© 2019 Nemeth B et al. *JAMA Network Open.*

eTable 3. Detailed List of All Surgical Procedures During Follow-up*

Type of surgery	1st surgery	All
**Orthopaedic**	-	
Knee arthroscopy	69	86
Cruciate ligament reconstruction	5	9
Minor orthopaedic (leg)	25	34
Minor orthopaedic (foot)	18	20
Minor orthopaedic (hip)	1	1
Major orthopaedic (leg/knee)	21	45
Major orthopaedic (foot/ankle)	1	2
Major orthopaedic (hip/pelvis)	30	45
Spinal disc	15	18
Spine	12	15
**Subtotal**	**197**	**275**
**Abdominal**		
Laparoscopy (peritoneal)	7	10
Cholecystectomy	34	37
Hernia (ing, fem, cic, epi, umb, spig)	45	71
Laparotomy	4	6
Pancreatic	0	1
Liver surgery	1	2
Minor abdominal	4	6
**Subtotal**	**95**	**133**
**Gastrointestinal**		
Oesophageal and stomach	8	16
Bowel	27	40
Appendectomy	9	10
Rectal	18	23
**Subtotal**	**62**	**89**
**Heart lung**		
Laparoscopy (thorax or mediastinum)	4	4
(hemi)thyroidectomy	3	5
Neck/cyst	3	3
Lung	7	14
Heart valve	3	5
Bypass heart, and other heart surgery	15	21
**Subtotal**	**35**	**52**
**Vascular**		
Endarterectomy	2	2
Major vascular (e.g. aorta)	9	12
Varicose vein	49	76
Arterial shunt	11	15
Small vascular	2	4
**Subtotal**	**73**	**109**
**Gynaecological**		
Ovarium surgery	10	13
Uterus surgery	35	41
Section Cesare	21	26
**Subtotal**	**66**	**80**
**Other**		
Surgical introduction central vein	2	3
Excision lymph node(s)	7	10
Major urology	4	5

© 2019 Nemeth B et al. *JAMA Network Open.*

Prostate surgery	1	2
Amputation leg	1	2
Breast surgery	28	33
Breast surgery, cosmetic	5	11
Brain surgery, skull	4	4
**Subtotal**	**52**	**70**
**Grand total**	**580**	**808**

* Surgeries classified in the abovementioned categories could also be classified in subcategories like major, minor, day care or cancer surgery. 1st surgery denotes the first surgery that was performed from start of follow-up. For example, 69 knee arthroscopies were performed as first surgeries, an additional 17 arthroscopies were performed following another primary type of surgery (thus 86 knee arthroscopies in total).

© 2019 Nemeth B et al. *JAMA Network Open.*

eTable 4. Absolute Risk of Recurrent Venous Thromboembolism After Surgery in Patients With a History of VTE (*Table of Figure 2 in manuscript*)

**Cumulative incidence, ci(t)**	**no. operations**	**Person years¶**	**no. VTEs¶**	**1-month** % (95% CI)	**3-months** % (95% CI)	**6-months** % (95% CI)	**1-year** % (95% CI)
**No Surgery**	-	18636	571	0.2 (0.1 to 0.5)	0.6 (0.4 to 1.0)	1.4 (1.0 to 1.9)	2.6 (2.1 to 3.3)
**Surgery (all)**	580	263	30	2.1 (1.2 to 3.6)	3.3 (2.1 to 5.1)	4.6 (3.1 to 6.6)	6.3 (4.6 to 8.7)
Major surgery	441	199	20	2.1 (1.1 to 3.9)	3.2 (1.9 to 5.4)	4.4 (2.8 to 6.8)	6.5 (4.5 to 9.3)
Minor surgery	198	92	8	1.5 (0.5 to 4.7)	2.6 (1.1 to 6.1)	3.6 (1.7 to 7.4)	5.2 (2.9 to 9.5)
Outpatient surgery	201	92	7	1.5 (0.5 to 4.6)	2.0 (0.8 to 5.3)	3.1 (1.4 to 6.7)	3.6 (1.7 to 7.4)
Cancer related surgery	44	21	2	4.7 (1.1 to 17.3)	9.3 (3.6 to 22.9)	9.3 (3.6 to 22.9)	9.3 (3.6 to 22.9)
**Orthopaedic surgery**	219	101	11	1.4 (0.5 to 4.2)	2.8 (1.3 to 6.1)	4.7 (2.5 to 8.5)	7.2 (4.4 to 11.7)
Major orthopaedic surgery	101	46	5	2.0 (0.5 to 7.7)	3.0 (1.0 to 9.0)	5.0 (2.1 to 11.6)	10.5 (5.8 to 18.6)
Minor orthopaedic surgery	135	63	5	0.7 (0.1 to 5.1)	2.3 (0.7 to 6.9)	3.8 (1.6 to 9.0)	5.5 (2.6 to 11.1)
**Non-orthopaedic surgery**	403	181	19	2.3 (1.2 to 4.3)	3.3 (1.9 to 5.7)	4.0 (2.5 to 6.5)	5.1 (3.3 to 7.9)
Abdominal surgery	120	55	6	1.7 (0.4 to 6.6)	2.5 (0.8 to 7.6)	4.3 (1.8 to 10.0)	6.1 (3.0 to 12.4)
Gastrointestinal surgery	73	32	6	4.2 (1.4 to 12.4)	7.0 (3.0 to 16.0)	8.4 (4.0 to 17.8)	10.0 (4.9 to 19.8)
Heart Lung surgery	46	21	2	2.2 (0.3 to 14.4)	4.4 (1.1 to 16.5)	4.4 (1.1 to 16.5)	4.4 (1.1 to 16.5)
Vascular surgery	89	40	3	2.3 (0.6 to 8.8)	2.3 (0.6 to 8.8)	2.3 (0.6 to 8.8)	2.3 (0.6 to 8.8)
Gynaecological surgery	73	35	0	Not estimable	Not estimable	Not estimable	1.5 (0.2 to 10.0)

Cumulative incidences were calculated from the day of surgery onwards.
* Patients at risk for recurrence with first operation, numbers (operation, person years and no. VTEs) do not add up because only the first operations during follow-up are included in the analyses (which can be all types of surgery depending upon surgery of interest).
¶ Person years and no. of VTEs only shown for the complete period of increased risk (0-6 months).

© 2019 Nemeth B et al. *JAMA Network Open.*

eTable 5. Association of Surgery With Recurrence Risk in Patients With a History of VTE*

VTE recurrence risk*	no. operations	Person years¶	no. VTEs¶	0 to 1-month HR_{adj}± (95%CI)	0 to 3-months HR_{adj}± (95%CI)	0 to 6-months HR_{adj}± (95%CI)	0 to 12-months HR_{adj}± (95%CI)
**No surgery (ref)**	-	18636	571	-	-	-	-
**Surgery**	580	263	30	6.8 (3.9 to 11.9)	4.2 (2.7 to 6.5)	3.5 (2.4 to 5.0)	2.6 (1.9 to 3.6)
Major surgery	441	199	20	6.6 (3.4 to 12.8)	4.1 (2.5 to 6.9)	3.1 (2.0 to 4.9)	2.6 (1.8 to 3.9)
Minor surgery	198	92	8	5.6 (1.5 to 14.2)	2.9 (1.2 to 7.0)	2.6 (1.3 to 5.3)	2.1 (1.1 to 3.7)
Outpatient surgery	201	92	7	4.7 (1.5 to 14.8)	2.4 (0.9 to 6.4)	2.3 (1.1 to 4.9)	1.3 (0.6 to 2.7)
Cancer surgery	44	21	2	4.5 (2.1 to 33.8)	4.6 (1.1 to 18.5)	2.7 (0.7 to 11.0)	2.0 (0.5 to 8.0)
**Orthopaedic surgery**	219	101	11	4.0 (1.3 to 12.4)	3.6 (1.9 to 7.5)	3.2 (1.8 to 5.9)	2.6 (1.5 to 4.3)
Major orthopaedic surgery	101	46	5	6.4 (1.6 to 25.7)	4.7 (1.8 to 12.6)	3.3 (1.4 to 8.0)	4.0 (2.1 to 7.4)
Minor orthopaedic surgery	135	63	5	2.1 (0.3 to 15.2)	2.5 (0.8 to 7.8)	2.4 (1.0 to 5.7)	1.9 (0.9 to 4.0)
**Non-orthopaedic surgery**	403	181	19	8.2 (4.4 to 15.3)	4.3 (2.5 to 7.2)	3.3 (2.1 to 5.2)	2.3 (1.5 to 3.6)
Abdominal surgery	120	55	6	5.0 (1.2 to 20.0)	2.7 (0.9 to 8.3)	3.0 (1.3 to 6.7)	2.3 (1.1 to 4.6)
Gastrointestinal surgery	73	32	6	12.3 (3.9 to 38.3)	7.3 (3.0 to 17.6)	5.1 (2.3 to 11.4)	3.7 (1.7 to 7.7)
Heart Lung surgery	46	21	2	8.2 (1.2 to 58.8)	6.3 (1.6 to 25.2)	3.3 (0.8 to 13.1)	1.8 (0.5 to 7.4)
Vascular surgery	89	40	3	7.1 (1.8 to 28.5)	2.6 (0.6 to 10.4)	2.2 (0.7 to 6.7)	1.2 (0.4 to 3.9)
Gynaecological surgery	73	35	0	Not estimable	Not estimable	Not estimable	0.8 (0.1 to 5.6)

*Only for first surgery exposure during follow-up, start follow-up is stop-date of anticoagulant therapy after first VTE.

±Hazard Ratio (HR_{adj}) adjusted for age & sex.

¶ Person years and no. of VTEs only shown for the complete period of increased risk (0-6 months).

© 2019 Nemeth B et al. *JAMA Network Open.*

eTable 6. Restriction Analysis of Absolute Risk of Recurrent Venous Thromboembolism After Surgery in Patients With a History of VTE, Excluding Patients With Cancer

**Cumulative incidence, ci(t)**	**Operations** number*	**1-month** % (95% CI)	**3-months** % (95% CI)	**6-months** % (95% CI)	**1-year** % (95% CI)
**Surgery (all)**	580	2.1 (1.2 to 3.6)	3.3 (2.1 to 5.1)	4.6 (3.1 to 6.6)	6.3 (4.6 to 8.7)
Surgery (all)†	533	2.1 (1.2 to 3.7)	3.4 (2.2 to 5.4)	4.8 (3.3 to 7.0)	6.7 (4.8 to 9.2)
Surgery (all)‡	478	1.9 (1.0 to 3.6)	2.7 (1.6 to 4.7)	4.3 (2.8 to 6.5)	6.3 (4.5 to 9.0)
**Orthopaedic surgery**	219	1.4 (0.5 to 4.2)	2.8 (1.3 to 6.1)	4.7 (2.5 to 8.5)	7.2 (4.4 to 11.7)
Orthopaedic surgery†	207	1.5 (0.5 to 4.4)	2.9 (1.3 to 6.4)	4.9 (2.7 to 9.0)	7.6 (5.7 to 12.3)
Orthopaedic surgery‡	200	1.5 (0.5 to 4.6)	3.0 (1.4 to 6.6)	5.1 (2.8 to 9.3)	7.9 (4.8 to 12.7)
**Non-orthopaedic surgery**	403	2.3 (1.2 to 4.3)	3.3 (1.9 to 5.7)	4.0 (2.5 to 6.5)	5.1 (3.3 to 7.9)
Non-orthopaedic surgery†	368	2.2 (1.1 to 4.3)	3.3 (1.9 to 5.8)	4.2 (2.5 to 6.8)	4.4 (3.5 to 8.3)
Non-orthopaedic surgery‡	317	1.9 (0.9 to 4.2)	2.2 (1.1 to 4.6)	3.2 (1.7 to 5.9)	4.6 (2.7 to 7.6)

† Exclusion of patients with cancer diagnoses within 5 years before first VTE (or shortly after).

‡ Additional exclusion of patients who developed cancer anywhere during follow-up.

© 2019 Nemeth B et al. *JAMA Network Open.*
